# Reconstructing a Meaningful Self: The Identity Work of People Living With Chronic Disease

**DOI:** 10.1177/10497323241303393

**Published:** 2024-12-10

**Authors:** Loraine Sonia Clur, Antoni Barnard

**Affiliations:** 1 Department of Industrial and Organisational Psychology, 56866 University of South Africa (UNISA), Pretoria, South Africa

**Keywords:** people with chronic disease (PwCD);, identity work;, meaning making;, existential reflection;, cognitive restructuring;, hermeneutic phenomenology;, multiple case study design

## Abstract

With the escalating number of people diagnosed with chronic disease globally, research aimed at supporting their adjustment and coping is invaluable. Reconstructing a sense of self is core to the psychosocial adjustment of people with chronic disease (PwCD), and meaning making is central to their coping with the diagnosis. Despite the growing number of PwCD living productive lives, their identity work is underexplored. This article reports on an in-depth multiple case study that explored the identity work of PwCD from a meaning-making perspective. Data were gathered from three cases using semi-structured interviews, document analysis, and diaries. Data analysis entailed interpretative phenomenological analysis and flexible pattern matching. Three themes describe participants’ identity work process: First, they narrate a broken identity, having experienced identity disruption, discontinuity, and loss; second, they envision an ideal identity through existential reflection; and third, they reconstruct a meaningful identity. Reconciling the broken self with an ideal self leads to the construction of a meaningful self. The meaningful self is conceptualized in participants’ application of Frankl’s meaning-making principles, as they constructed a purposeful self (creative), a connected self (experiential), and a determined self (attitudinal). The article discusses the implications for helping professionals and organizations in supporting PwCD as they work toward rebuilding a meaningful self, facilitating their identity work in the search of a meaningful self.

## Introduction

It has been reported that a third of the global adult population lives with one or multiple chronic diseases and the number continues to increase (Hajat & Stein, 2018; Shelton et al., 2022). Chronic disease (CD) is a permanent health condition which is objectively diagnosed based on a clinical symptomology (Samwiri Nkambule & Msiska, 2024). To better manage the consequences of CD, a body of research focuses on the study of chronic illness, which refers to the subjective experience of living with CD (Fuller, 2018; Samwiri Nkambule & Msiska, 2024). Such a person-centered perspective contributes to holistic and multidisciplinary support for people with CD (PwCD), as it focuses on the person’s experience and perceptions of their CD and how they cope with and adjust to it (Schwarz et al., 2024). The importance of research on chronic illness continues to escalate globally, as it goes beyond the prevention and management of CD to incorporate a perspective on supporting PwCD to live and work well within the context of their disease (Robinson, 2017).

Living with a CD entails physical and psychological challenges that have a disruptive effect on people’s general and occupational functioning (Bosma et al., 2020; Kagura et al., 2023; Varekamp et al., 2013). Central to the experience of these challenges is a fundamental reshaping of one’s identity (Beatty & McGonagle, 2018; Schwarz et al., 2024). A CD diagnosis disrupts the sense of self and prompts a critical assessment of one’s own significance (Filej et al., 2018). This leads to reconstructing one’s identity to cope with and adjust to living with the CD (Hoffman et al., 2013; Van Deurzen, 2014; Walder & Molineux, 2017). The process of identity reconstruction, theoretically referred to as identity work (Brown, 2021; Caza et al., 2018), encapsulates a processing of the self to cope with identity threat (Breakwell, 2010) and retain a congruent sense of self (Rogers, 1961). Identity resilience is achieved when a person constructs a relatively congruent identity structure that facilitates adaptive coping and enables functional adjustment to life’s challenges (Breakwell, 2021).

The effect of CD is incremental, leading to chronic illness that affects identity in various ways over the adult life span (Beatty & McGonagle, 2018). While Christiansen (1999) argued that identity is directly related to what one does and primarily expressed through work, Beatty and McGonagle (2018) note the importance of viewing work as central to the identity of PwCD and essential to their adjustment process. For PwCD, work provides normality, an income, meaning and purpose, a sense of belonging, and enhanced self-worth (Leonardi & Scaratti, 2018; Vooijs et al., 2018). Consequently, a substantial body of occupational health research has highlighted that reconstructing an occupational identity facilitates the adjustment process for PwCD (Walder & Molineux, 2017). In this study, we take the view that occupational identity is an important part of an individual’s overall identity development and consider participation in work to be an important defining aspect of PwCD’s identity. While research generally confirms identity threat, disruption, and change as an inevitable consequence of chronic illness and identity reconstruction as essential for positive adjustment (Beatty & McGonagle, 2018; Schwarz et al., 2024; Walder & Molineux, 2017), scant research has focused on describing the process of identity work. Santuzzi and Waltz (2016) emphasize the need for continued research to enhance understanding of the complexity of PwCD’s identity and the way it changes. Beatty and McGonagle (2018) propose mechanisms to explain the identity change process in PwCD, while calling for further, more detail-specific research in this regard.

Apart from the need to reconstruct one’s identity, chronic illness research has shown that when people have to live with a CD, making meaning is a critical coping strategy for positive adjustment and well-being (Hartog et al., 2020; Martino et al., 2019; Winger et al., 2016). Meaning making is also closely tied to identity and identity work. Identity gives meaning to life experiences such as chronic illness (Walder & Molineux, 2017) and provides the context for making sense of life and deriving meaning in life (Brown, 2021; Christiansen, 1999). Guided by the question “How do PwCD maintain a congruent sense of self?”, the objective of the study was to explore the identity work of PwCD from a meaning-making perspective.

## Literature Review

Two main theoretical streams informed the development of the theoretical propositions that guided data analysis and were applied as an interpretive lens to make meaning of the data (Kivunja, 2018; Varpio et al., 2020), namely, identity and identity work, and meaning making.

### Identity and Identity Work

Identity refers to the subjective meaning that a person ascribes to the self, based on their personal attributes, group memberships, and the roles (such as work role or work identity) they hold (Brown, 2021; Caza et al., 2018; Stets & Serpe, 2013). The multiple elements of self-meaning constituting one’s identity are structured according to how important and desirable they are to the individual (Breakwell, 2010; Brenner et al., 2014). Identity elements that are deemed psychologically central to, important for, or prominent in one’s sense of self represent those self-meanings that are highly valued by the individual (Brenner et al., 2014) and reflect the ideal self or identity standard that people strive to uphold (Burke & Stets, 2023). The ideal self is socially constructed and based on one’s normative and socialized understanding of self–other expectations. The need for identity congruence thus motivates behavior because people want to sustain congruence between the experience of self and their perception of how they ought to be (Burke & Stets, 2023; Rogers, 1959, 1961). People feel authentic and their best self when they act in ways closely related to the ideal self (Jacobs & Barnard, 2022). A congruent identity enables people to uphold self-continuity, self-efficiency and self-worth (Breakwell, 2010), self-esteem (Stets & Burke, 2014), motivation (Sveningsson & Alvesson, 2003), work engagement (De Braine & Roodt, 2011), and well-being (Bauer et al., 2008; Haslam, 2014; Waterman, 2007) and enables adjustment and coping (Barnard & Flotman, 2020).

Transformative life events (such as CD) pose a threat to one’s identity, challenging one’s ability to maintain a sense of efficacy, belonging, and self-worth (Breakwell, 1988; Brygoła, 2011). This threatens the experience of identity continuity (Brown, 2021) and leads to experiencing self-incongruence (Rogers, 1959). In response, individuals engage in identity work to modify their identity so that it continues to sustain their self-esteem and validate their worth in society (Petriglieri & Stein, 2012). Identity work encompasses various ways of crafting, revising, sustaining, enhancing, or modifying one’s meaning of self in order to produce a sense of self-coherence (Alvesson & Willmott, 2002; Sveningsson & Alvesson, 2003) or congruence with the ideal self (Jacobs & Barnard, 2022; Rogers, 1961). Identity work is a natural response to coping with difficult life demands (Pals, 2006), and it invokes the psychological processes of self-evaluation and self-verification which manifest in thoughts, behavior, and emotions (Breakwell, 2010; Caza et al., 2018) both consciously and unconsciously (Petriglieri & Stein, 2012).

On receiving a CD diagnosis, PwCD experience a threat to their identity (Breakwell, 1988), and through self-evaluation and self-verification they will try to maintain their identity standard and support self-congruence as proof of their continued self-worth. When the diagnosis causes self-evaluation to result in a sense of identity non-verification, negative psychological consequences such as anxiety and depression may prevail (Burke & Stets, 2023). Self-verification enhances self-esteem, while identity non-verification has a negative effect on self-worth (Brenner et al., 2018). In pursuing self-verification, PwCD may also engage with the identity work process of assimilation to incorporate newly valued identity elements and reconstruct the self (Brygoła, 2011), thus reducing identity tension and regaining self-congruence (Rogers, 1959).

### Meaning Making

Existential psychology focuses on how a person can find or create meaning in order to adjust and function well in the context of challenging or disturbing issues of existence (existential dilemmas) (Yalom, 1980). Meaning making from an existential perspective entails a consistent processing of how the self, in relation to others and events, fits into the greater context of life (Gustavsson, 2018). It incorporates existential questions like “Who am I?” and “What is my purpose in life” (Reker, 2000, p. 39). Individual responses to these questions imply the constant study of one’s meaning in the world and a reflection on one’s sense of self in searching for self-fulfillment (Joshi et al., 2023). People living with a CD are confronted with existential dilemmas and experience existential crises (Bachhuber, 2011) or existential anxiety, which is described by Frankl (2008, 2019) as an existential vacuum or a sense of meaninglessness. People who do not resolve their existential anxiety experience a sense of inauthenticity (Temple & Gall, 2018) and a sense of self-detachment or non-being (Wong, 2016). When confronted with existential anxiety or identity conflict, people attempt to cope and regain a sense of authenticity by engaging in a process of meaning making (Ownsworth & Nash, 2015), which according to Christiansen (1999, p. 552) happens through “selfing” or, in the context of this study, through identity work.

Viktor Frankl, a pioneer in the study of meaning, constructed meaning-making principles from his existentialist philosophy and difficult personal life experiences (Bushkin et al., 2021; MacKenzie & Baumeister, 2014). Despite Frankl being seen as an existentialist, he also embraced a cognitive perspective, recognizing that people interpret life events and make decisions about them cognitively (De Klerk, 2006). From a cognitive meaning-making perspective, individuals are information processors influenced by their social context. They process life events to self-regulate and uphold self-continuity (Berzonsky, 2011). The existential approach to meaning making comprises *reflection* (Wong, 2017), whereas the cognitive approach entails *restructuring* (Osin et al., 2016) of the self.

Ultimately, meaning making is always directed toward fulfilling four basic needs: to experience the self as having purpose, efficiency, justification, and worth (Christiansen, 1999). Frankl (1988, 2008) posits that such meaning is derived from creative, experiential, and attitudinal values. The creative pathway of meaning making involves engaging with deeper awareness or purpose (Pattakos & Dundon, 2017) by creating a work or performing a deed (Frankl, 2008). The experiential path comprises meaningful connectedness through relationships or encounters with others (Frankl, 2008; Pattakos & Dundon, 2017), as well as with nature, beauty, and other aspects of life (Wong, 2016). The third avenue to meaning in life, the attitudinal pathway, reflects the way one approaches life (Frankl, 2008). Frankl’s three enduring values—creative, experiential, and attitudinal—effectively capture the three pathways to reconstructing the self and finding meaning in life (Wong, 2014).

## Methods

### Study Design

Guided by the features of case study design (Creswell, 2013; Yin, 2014), a qualitative multiple case study was conducted. The case study applied within-case and cross-case analysis of three real-life cases in which persons with longstanding CD diagnoses continued to participate in work despite the persistent physical and psychosocial challenges of their conditions. The cases were instrumental (Stake, 1995) in that the intent was to explore how participants maintained a congruent sense of self within the context of living and working with a CD. Cases were thus selected to provide insight into and identify patterns of identity work from their lived experiences rather than understanding the particulars of each case (cf. Holten et al., 2018). Accordingly, the study findings report case themes to describe patterns of identity work (Creswell, 2013). Over a period of 3 months, multiple data sources were used to gather qualitative data on each case to enhance an in-depth understanding of the cases (Creswell, 2013). The strategy chosen for data analysis combined the phenomenological principles and analytical steps of interpretative phenomenological analysis (IPA) (Eatough & Smith, 2017; Pietkiewicz & Smith, 2014) for the within-case analysis, together with a deductive flexible pattern-matching technique (Bouncken et al., 2021; Sinkovics, 2018) for the cross-case analysis. This dual analytical strategy follows that of Holten et al. (2018) who, in their multiple case study design, combined constructivist grounded theory for within-case analysis with the pattern-matching method for cross-case analysis.

### Theoretical Orientation and Researcher Positionality

The researchers subscribe to a hermeneutic phenomenological orientation, recognizing knowledge generation as vested in the interpretation of subjective lived experiences (Boden & Eatough, 2014; Suddick et al., 2020; Tomkins & Eatough, 2018). From this orientation, interpretation is influenced by researchers’ positionality and theoretical preconceptions (Suddick et al., 2020) and findings are offered as a contribution to the body of knowledge rather than being the only interpretations available (Boden & Eatough, 2014). It is thus appropriate to acknowledge that this study was influenced by the researchers’ occupations, personal experience with chronic illness, and proclivity for meaning making and identity work as important mechanisms for coping and adjustment. As a person living with multiple chronic diseases, the primary researcher (LC) supports PwCD in her private practice through coaching and counselling. Consequent to her experience of coping with CD, and through her master’s and doctoral studies, she has developed a practice approach informed by existential meaning-making theories to support PwCD in their coping and adjustment. The secondary researcher (AB) is an industrial psychologist registered with the Health Professions Council of South Africa and an academic with a particular research interest in the identity work of people who are coping with and adjusting to uniquely challenging work–life contexts. The researchers’ relationship started over a decade ago when AB first supervised the master’s and then later the PhD of LC. This study is based on data from LC’s PhD research and the findings reflect the “fusion of horizons” (Suddick et al., 2020, p. 3) typical of the hermeneutic phenomenological approach of both the researchers and the participants.

Congruent with the above, case study design allows for case analysis within the context of existing theory (Crowe et al., 2011; Yin, 2014). Guided by the case study method of Yin (2014), this multiple case study followed a theory-first approach and applied the principle of the hermeneutic circle (Tomkins & Eatough, 2018). The theory-first approach entailed deriving theoretical propositions from the literature review to guide data analysis (Holten et al., 2018). Hermeneutic circling guided interpretation in that it was used to move between and compare parts and whole—for example, within-case and across-case analysis—while constantly comparing data and theory. Hermeneutic circling entails inductive and deductive thinking as mutually constitutive sense-making processes (Tomkins & Eatough, 2018). This allowed the application of IPA during within-case analysis together with a flexible pattern-matching method for cross-case analysis, still within the broader context of a theory-first case study. IPA highlights the centrality of lived experiences and aims to inductively explore the processes through which people, as self-reflective beings, make sense of the challenges and difficulties they experience in their lives (Brocki & Wearden, 2006; Pietkiewicz & Smith, 2014; Smith, 2018). It is well suited to case study research and, within a hermeneutic phenomenological frame, IPA can still be informed by theoretical preconceptions (Eatough & Smith, 2017). The cross-case analysis followed, as well as applied flexible pattern matching (Bouncken et al., 2021), to deductively use a priori theoretical propositions to guide the construction of themes.

Theoretical propositions guiding interpretation in this study were developed from the literature review and are summarized as (i) PwCD experience an existential crisis disrupting their sense of self; (ii) PwCD are innately driven to make meaning of their situation to maintain a congruent sense of self. To maintain a congruent sense of self, PwCD engage in the processes of identity work, namely, self-evaluation, self-verification, and assimilation, through reflection and cognitive reconstruction; (iii) in the process of identity work, people revise their self-meanings in line with a self-constructed identity standard to cope with and adjust to living with CD; and (iv) in the process of identity work, PwCD apply the creative, experiential, and attitudinal pathways as meaning-making strategies to revise their sense of self.

### Selecting the Cases and Collecting Data

Case selection was based on a purposive and convenient sampling approach. Purposive sampling included cases (i) with a formal CD diagnosis; (ii) living with the CD for at least 10 years or more; and (iii) being economically active during this time. From a convenience perspective, potential participants were contacted by the primary researcher from a support network for PwCD to which she belongs. Cases were homogeneous in that they all shared the perspective of living and working with CDs for over 14 years, were within the age group 40–62, lived in South Africa, and spoke Afrikaans. Two females and one male diagnosed with various CDs (alopecia areata, ankylosing spondylitis, asthma, hypertension, breast cancer, and psoriasis) participated. Pseudonyms are provided for individual cases (Fiona, Dwayne, and Dinah) to guard their anonymity and enhance confidentiality.

Data were collected from multiple sources, including semi-structured interviews, follow-up discussions via WhatsApp, telephone and email, document analysis, and diaries. Initial semi-structured interviews were conducted based on five pre-prepared questions (“Tell me about your chronic illness(es) and the role it plays in your life.”; “What does a typical day living with a CD look like?”; “What are your ways of coping with your CD?”; “How do you make meaning while living with chronic conditions?”; and “What gives meaning to your life?”). Probing questions developed naturally during the interviews and more information regarding their chronic illness and work and the workplace emerged. One participant gave access to a published autobiographic article as an important data source. After asking their permission during initial interviews, participants were engaged in follow-up communication via email, WhatsApp, and phone to further probe and clarify emergent aspects. Following discussion in the initial interviews, two participants agreed to record their daily experiences in a diary for a week. In doing so, one participant employed art and poetry to express experiences, which supplemented the data with rich visual metaphorical descriptions.

### Ethics

Ethics clearance was obtained from the relevant UNISA Health Research Ethics Committee (2021/CAES_HREC/015). Participants gave written informed consent. Owing to the small sample and the intimate nature of narratives, confidentiality was maintained by using pseudonyms, by changing or omitting personal or contextual identifiers in the data and ensuring that the data were only accessible to the researchers and securely stored in password-protected e-formats. The participants were protected from harm by discussing with them their freedom to withdraw and their access to counselling (McCosker et al., 2001) prior to participation. During the interviews, this was confirmed, noting that if they felt any emotional discomfort, the option to end the interview and be referred for counselling was open to them. However, this was not needed during any of the interactions with the participants. During data collection, the primary researcher maintained empathy and established rapport by acknowledging her CD and her interest in studying PwCD. To support her ability to remain open-minded, she did not participate in this study but took the role of an insider–outsider researcher (Dwyer & Buckle, 2009) and employed self-reflection. She kept a journal documenting her experiences and thoughts during the study, and the researchers conducted regular debriefing discussions to facilitate awareness of biases and subjectivities, as well as to explore and question research decisions and interpretations.

### Data Analysis

As demonstrated by Holten et al. (2018), in multiple case studies, every case is individually and separately analyzed before making comparisons across cases (Crowe et al., 2011). Whereas Holten et al. (2018) applied grounded theory analysis for the within-case analysis, this study followed the analytical stages of IPA as proposed by Pietkiewicz and Smith (2014). First, the interviews for each case were transcribed, and all related written material was organized in a data set per case. Second, the data set for each case was read multiple times while making initial notes. Third, notes were reviewed for similarities across the data sources per case and transformed into possible themes. Fourth, themes were condensed and integrated by grouping related themes and giving them descriptive labels. The themes generated from this within-case analysis were informed by the literature on identity theory and meaning making but did not yet provide a meaningful condensed thematic structure across all three cases or clearly relate to the study propositions. Like Holten et al. (2018), the study consequently applied pattern matching for the cross-case analysis. Specifically, the cross-case analysis followed the flexible pattern-matching method described by Bouncken et al. (2021). The flexible pattern-matching process entailed comparing the emergent themes from the within-case analysis with preconceived theoretical propositions. The lens of the propositions facilitated the grouping, condensation, and conceptualizing of themes to allow for the construction of the final themes (Bouncken et al., 2021; Sinkovics, 2018).

### Quality

Strategies for rigor and trustworthiness followed Yardley’s (2000) principles for ensuring quality research and Creswell’s (2013) defining features for case study inquiry. The researchers’ sensitivity to context was demonstrated by a thorough, focused review of relevant literature and the use of multiple data sources to enhance a deep understanding of each case. Methodological rigor was enhanced by multiple readings of the data set for each case. Combining the analytical methods of IPA and the flexible pattern matching facilitated a thorough and structured analysis. This also enhanced the application of hermeneutic circling, which involved constantly comparing within-case data, cross-case data, and literature. Using a priori theoretical propositions added reliability to the findings (Holten et al., 2018) and ensured the construction of (i) a thematic structure demonstrating theory–data congruence and (ii) a coherent narrative based on a fusion of case data and researcher interpretations. The primary researcher kept a reflexive journal, noting her thoughts, emotions, interpretations, and observations throughout the research process. In debriefing meetings with the co-researcher, potential transferences and projections that may have influenced participant engagements, data collection, and analytic decisions were discussed and addressed as necessary. In regard to the impact and importance of the findings (Yardley, 2000), recommendations are noted for PwCD and healthcare professionals who are coaching, consulting, or counselling the former in the adjustment and coping process, as well as for organizations employing PwCD.

## Findings

Three themes were constructed. First, when confronted by the CD diagnosis, participants experienced identity disruption, discontinuity, and loss, conceptualized as a broken self. Second, participants sought identity congruence through existential reflection, forming an ideal self. Third, participants engaged in cognitive reconstruction of a meaningful self.

### Broken Self: Experiencing Identity Disruption, Discontinuity, and Loss

When diagnosed with a CD, the participants’ identities were notably disrupted by changes to their physical appearance. Self-evaluation led to identity non-verification, reflected in the experience of identity incongruence and an overall sense of identity loss. In all three cases, the identity loss was an embodied experience. Fiona’s hair loss invalidated her past self-meanings. Her loss of self is evident in the way she identifies with and speaks about her hair:

Hair is seen through the ages as a symbol of femininity, sensuality, and beauty but more than that, it gives you an identity. My whole life, my hair was my joy, and I can honestly say that I never had a bad hair day … A deep pain lodged in my body and, believe me, for a long time, I could not look at myself in the mirror without experiencing pain … I felt naked. It was the start of a long mourning process.

Dwayne experienced identity disruption and loss in the context of joint pain and an altered physique. He remembered how he felt invalidated by his experience of how others responded to him and how he consequently started weightlifting to feel accepted again:

Sometimes one gets mocked by people … laughed [at] … I started to walk funny. To this day, I am still teased by people for the way I walk … I worked really hard because I wanted to appear like a weightlifter. I feared that people would think I didn’t look the part.

Dinah’s embodied loss of self was evident in her experience of breast cancer and psoriasis. She referred to the “loss of a breast” and “loss of sexuality” on numerous occasions. In her diary, she drew a picture, labelling it “Storm.” She used it to describe herself as “fragmented, broken horizon, known in part.” In a follow-up discussion, it became clear that her experience of a fragmented broken self, resembled identity non-verification and discontinuity, as she reflected that the drawing resembles “a representation of one’s own brokenness, whether it’s illness, work, or death, which you don’t always understand. The incomplete understanding of it, or its brokenness, is depicted as a whirlpool, a whirlwind.” The experience of not understanding herself fully anymore indicates her loss of self and made her feel depressed and angry: “I should just add that a lack of comprehension frequently leads to anger.”

The physical consequences of the CD and how it affected interactions with others led to the experience of identity discontinuity and loss, as a real, embodied experience that left participants feeling not only physically but also psychologically broken. Feeling broken translates to a sense of self-incongruence and elicits existential reflection, seeking self-continuity and a regained sense of self-congruence.

### Ideal Self: Negotiating Identity Continuity Through Existential Reflection

Participants’ identity work was evident in a reflective self-verification process that involved consistent comparison of the self in regard to who they were (past), who they are (present), and who they wanted to be (future). The comparative self-verification entailed negotiating identity continuity to regain a sense of self-congruence (Baker et al., 2018; Zafran et al., 2012). Fiona’s existential reflection on how the CD had changed her identity from past to present is poignant: “I had to dig deep in my inner being to seek for that which made me more than the person which I knew until then, the one with the beautiful hair.” In the existential reflection, the question “Who am I now?” seems central in dealing with the broken self and, in their reflection on this question, the participants created positive versions of how they would like to be perceived—a desired or ideal self.

The ideal selves of the three participants are metaphorically illustrated in the data through the animated characters with whom they explicitly identified. The character of Fiona from the film *Shrek* (Jenson & Adamson, 2001) was chosen to portray Fiona’s ideal self as an adaptable, empowered, and strong-willed individual. She said that she did not want to feel like a “… tiny baby bird that has fallen out of its nest … Anyone who knows me well would attest to the fact that I am not a submissive woman, hair or no hair, scarf or no scarf. I’m too free for that.” In expressing her ideal self, Fiona wanted to be respected for being self-sufficient and self-determined and she wanted to continue “exploring the wider world and seeing me as part of the larger scheme of things.”

From the animated film *Toy Story* (Lasseter et al., 1995), Dwayne identified with the archetypal hero character, Buzz Lightyear, expressing his desire to be a positive role model. In the film, Buzz gradually accepts that he is a toy rather than a spaceman destined to save the universe. In doing so, he becomes a different type of hero—lesser in stature but ultimately greater as he becomes more focused on others (Placido, nd). Dwayne stated as follows:

It is important for me to be a positive role model … I believe that I might be overcompensating a little for the disadvantage by constantly attempting to be stronger, smarter, fitter, or more diligent than the typical individual … I want to be the best relative to others. I constantly want to prove the opposite. I want to defy the status quo by being the most active AS person, an asthmatic who can exercise in a gym and do cardiovascular activities.

In negotiating identity continuity, Dwayne wanted to feel strong and self-sufficient, and the acceptance and validation of others was essential to enable him to feel worthy. In his words, he was motivated by the ideal he aspires to: “I know that my looks and athleticism are not the best, so I can just better myself by working harder*.*”

Dinah identified her ideal self with Lisa from the animated television series *The Simpsons* (Groening, 1989). Dinah expressed her ideal self as wanting to be seen to be “making a difference” and “to mean something to another human being,” comparing herself to the character of Lisa, who is kind and compassionate (Lisa Simpson, nd). Dinah’s initial identity non-verification led her to construct an ideal self as someone who finds meaning through helping others. Her constant self-evaluation toward this ideal self is reflected in her narrative:

After the cancer, the corporate world was meaningless ... It did not make any sense anymore. There is this enormous void because I just don’t mean anything. I so miss being able to give something to someone, and it shouldn’t be my mother, it shouldn’t be my child, it shouldn’t be my husband.

In reflecting on their broken selves, the participants began to make meaning of their illness experiences by expressing identities aligned with a self-constructed identity standard, expressed as ideal selves. The participants’ ideal selves were particularly reflected as being self-sufficient and self-determined (Fiona and Dwayne), being regarded as worthy (Dwayne), and having meaning and purpose (Dwayne and Dinah). To embody their ideal selves, participants engaged in reconstructing their identities through Frankl’s (2008) meaning-making pathways.

### Meaningful Self: Reconstructing Identity Through Meaning-Making Pathways

From a cognitive perspective, PwCD’s identity work is aimed at making sense (meaning) of their situation (Harrison & Tronick, 2022) and ensuring self-congruence and identity continuity (Erikson, 1994). To do so, they applied Frankl’s (2008) creative, experiential, and attitudinal pathways to construct a meaningful self.

Participants’ application of the creative pathway was evident in their efforts to reconstruct meaningful work identities. They constructed a meaningful self as someone who engages in more purposeful and creative activities, which provided them with meaning and enabled them to assimilate their broken selves into their identities, reconciling them with their ideal selves. Both Fiona and Dinah applied the creative pathway by leaving the formal work sector to become entrepreneurs, focusing on creating art and doing purposeful work. Fiona noted how her art gave her meaning: “This is now my real work and I enjoy it so much that it does not feel like work. I lose myself in it and can paint for hours on end without getting tired … it gives me energy, calmness and is intensely satisfying.” It would appear that she has found her calling, as she states that “I work to live and do not live to work … work never defined me as a person. I will never miss it.” Dinah similarly craved more purpose and meaning in her work: “I decided, I’m not going to stay here any longer. It was a conscious decision to choose a better life. The job no longer made sense … It had been a chase after money.” She subsequently did a counselling course to pursue her ideal self as wanting to mean something to others: “Understanding how people’s inner beings worked makes me happy … That is why my work was meaningful to me … it excited me and gave me purpose. The [volunteer] work means something to another human being. I feel my world go around.” Dwayne’s application of the creative pathway in creating a meaningful self was evident in his active pursuits, such as teaching youngsters at church and assisting people at work. These activities made him feel appreciated, valued, self-sufficient, and worthy:

I get frequent compliments from my manager concerning the tasks that I complete quickly and on my problem-solving skills. I think my colleagues trust me because they do not hesitate to ask my advice or seek my counselling. People at work will say of me, “He just goes on, he has his finger on the pulse. If he feels compelled to do something, such as raising the desk, he does so. He doesn’t need anyone’s pity or assistance.” I believe they would say that I do not want to be given special treatment, I can take care of myself.

Participants’ application of the experiential pathway was evident in their reconstruction of meaningful relationships. Constructing a connected self helped them to assimilate chronic illness into their life and thus enhance a sense of self-congruence. Fiona expressed a strong connection with her husband, friends, and colleagues, recalling that “loneliness was not part of the process” because “my husband always holds me … I have people to love and who return the love … I worked with a wonderful team of women, and it was an ultimate enriching ten years of my life.” Fiona’s travels also brought her a sense of connectedness: “It’s nice to be able to travel, and also to reside in other countries, because for me to remain in one country for a lifetime, would have been very limited*.*” Dwayne’s identity was rooted in family, saying that “I had some leave and spent it at home doing fun things with the kids … There is no better place as home.” Connections at church, work, and the gym also gave him a sense of meaning and belonging: “It [teaching children at church] gives me meaning and balance” and “I get frequent remarks like, ‘if you want something done, ask Dwayne,’” and “An older lady at the gym also said that I inspire her because she sees that I have difficulties bending over, but in spite of that, I always work the hardest.” Lastly, Dinah’s connection with a friend supported her feeling connected when she was at her worst: “My friend was really close to me at the time and helped me a lot. I literally pushed myself to do things despite the fact that I felt like I was about to die. I once sat in a corner and cried because the loneliness was unbearable. I then called my friend, who arrived at the hospital and bathed me.” She recalled a connectedness with colleagues when returning to work: “Everyone was overjoyed to see me, and I was overjoyed to see them. It felt like a family reunion.” To reframe her connected self when she resigned, Dinah continued to do volunteer work to help other cancer patients.

Participants applied the attitudinal pathway in order to construct the self as being positive and self-determined. By changing their attitude, they assimilated their broken selves into their ideal selves, into their identity. Fiona’s positive mindset was expressed through humor, saying that without hair she could now frequently have “a good hair day” and that focusing on “the good in life far outweighs the negative.” She emphasized her agency and choice by describing how she changed her attitude: “reminding myself to not take anything for granted, to not compare myself with others, am slower to judge, to not waste energy on false hope and discouragement and choose to laugh rather than to cry.” Dwayne adopted a positive attitude and expressed self-determination by assimilating chronic illness into his ideal self as a weightlifter. He expressed this as follows:

I want to be the first guy to beat AS or who can exercise himself out of a blood pressure problem. God addresses our attitude … we are placed in this world to make an impact on others … so remain strong. I motivate myself by proving everyone incorrect who claims that something cannot be done. So, this is a way for me to push myself by showing the naysayers that, despite having AS, I can still squat and deadlift 150 kg.

Dinah’s motivation to construct a meaningful self is evident in the way she reorientated her attitude to assimilate chronic illness into her identity and pursued her ideal self of being meaningful to others. She noted:

Our minds determine who we become … I continue to work like a maniac. I’m really trying. It’s quite pleasant for me to occasionally help people with CD, it makes me happy … Even if you fail a thousand times, remember that if you are willing to look past the cuts and hard work, the broken glass shards of your life will eventually form an awesome mosaic.

## Discussion

The aim of the study was to explore the identity work of PwCD, applying the theory of meaning making as a theoretical lens. The findings reveal that participants’ identity work progressed through three phases: the broken self, the ideal self, and the meaningful self. Throughout all three phases, participants engaged in the identity work processes of self-evaluation, self-verification, and assimilation to make meaning of the self in the context of CD. This suggests that the three identity work phases did not occur in a linear or sequential way; rather, they were iterative, as PwCD consistently apply self-evaluation, self-verification, and assimilation to reconcile their broken and ideal selves into a meaningful self.

In the first phase, following their CD diagnosis, self-evaluation led to a sense of identity non-verification during which participants identified a broken self, characterized by a disruption in the meanings they had previously ascribed to the self. This self-non-verification inadvertently triggered negative emotions such as depression and anger, culminating in an overall sense of loss of self. The concept of a broken self aligns with other studies that highlight that people suffering from CD experience self-loss (Golub et al., 2014), radical identity changes (Morley, 2022), and the need to repair their self-identity (Charmaz, 1995, 2016).

During the second phase, participants’ responses to the disruption and loss of identity (i.e., the broken self) involved existential reflection, as they sought to answer the question “Who am I now?” While existentialists emphasize the need to engage in meaning making by considering existential questions relating to the self (see Bachhuber, 2011; Ownsworth & Nash, 2015; Temple & Gall, 2018), our study related such existential reflection to identity work processes. The existential reflection was driven by an ongoing need for self-verification in the context of felt incongruence resulting from the broken self. To resolve this identity tension, the participants needed to rediscover existential meaning. Their quest for self-verification indicated a strong desire to reconcile their broken selves with a more positive sense of self—one more akin to who they were before the onset of the CD and one more aligned with their self-perceived identity standards.

In the process of resolving the identity incongruence, their identity work involved expressing an ideal self, through which they voiced their desires for how others should perceive them and sought an identity that would mask the deficiencies associated with their CD. Their desired positive definitions of self as self-sufficient, worthy, and meaningful culminated in the portrayal of an ideal self.

Developing the ideal self was, however, insufficient for maintaining self-verification, because it reflects a future, desired sense of self which needs to be reconciled with the broken self. The ideal self is formed through existential reflection as a response to being constantly challenged by experiences of a broken self. Participants thus engaged in a third phase of self-reconstruction to assimilate their broken selves, thus reconciling the broken self with the ideal self. The ideal self remains tentative and searching in the shadow of the broken self, whereas the meaningful self portrays a more established and enacted sense of the desired “who I am.” Making meaning through identity work in this way entailed integrating the CD into their positive sense of self and led to constructing and enacting a meaningful self. During this third phase of identity reconstruction, participants’ identity work could be conceptualized through the application of Frankl’s (2008) meaning-making pathways. The meaningful self, from the perspective of Frankl’s meaning-making principles, is thus conceptualized as a processual concept, as it is based on the ongoing search for meaning and purpose (through the creative pathway), and staying connected (through the experiential pathway) by striving to be positive and self-determined (through the attitudinal pathway).

Participants’ identity work following their CD diagnosis thus entailed an adjustment process that commenced when becoming conscious of their broken selves and was followed by expressing an ideal self through existential reflection and reconstructing a meaningful self. Ultimately, in negotiating the tensions and imbalances between the broken and the ideal self, the striving is for meaning and the reconstructing and maintaining of a meaningful self. Based on Frankl’s meaning-making principles, the meaningful self is conceptualized as one who is purposeful in what they do, is connected to significant others and the outside world, and is self-determined and positive in their orientation to life. Identity work is thus represented as a continuous meaning-making process with the purpose of maintaining self-congruence.

## Strengths and Limitations

A theory-first approach applying theoretically grounded propositions provides a strong research design, as well as focused data analysis (Holten et al., 2018). It may, however, limit the phenomenological focus in that it reports on individual cases and reflects researcher bias in the form of applying predetermined interests and assumptions to the research project and the data. The inductive–deductive approach of this study, within the guiding context of theoretical propositions, applied a dual analytical strategy for within-case and across-case analysis in order to enhance researcher sensitivity to the experiences and voices of individual cases. Reflection strategies (journaling and researcher debriefing discussions) were applied and, in acknowledging the researchers’ positionality and predispositions, the study does not claim absolute truth but offers the findings as a contribution to the fields of PwCD research and identity theory. The study was strengthened by the richness of the data gathered from multiple sources over a period of 3 months (Creswell, 2013). The small number of cases used to explore the experiences of various CDs may, however, have limited the representative richness of the data for people with different CDs. Nevertheless, the focus of the study was not on a specific CD experience but on understanding the cases’ identity work processes to offer pragmatic guidance to PwCD, as well as to the healthcare professionals supporting their adjustment and coping.

## Implications and Recommendations

In comparing the three cases, meaning-making theory was applied to construct knowledge about the identity work of PwCD, promoting an awareness of the intimate and challenging self-processing that is central to adjusting to and coping with the effects of living with a CD. The study provides individuals with CD, as well as health professionals, with a unique understanding of the self-adjustment process that PwCD face. Apart from increased self-awareness for PwCD, the findings of this study could strengthen health professionals’ practical approach to facilitating the identity work of PwCD in order to develop a meaningful self. To support identity work in the pursuit of a meaningful sense of self, PwCD’s reflective sense-making and adjustment should focus on (i) *repositioning* their work purpose (the creative pathway); (ii) *remaining* connected (the experiential pathway); and (iii) *reframing* their life orientation (the attitudinal pathway). By doing so, PwCD can enjoy greater personal fulfillment, as well as experiencing a positive impact on their general coping and adjustment.

The role of work forms a big part of people’s lives and the organization should be prepared to perform its vital role in supporting PwCD in their reintegration in and adjustment to the workplace. Understanding how a person processes the self to make meaning while living and working with a CD provides valuable information for supporting the adjustment and coping of PwCD, as well as for employers to better accommodate, support, and retain such employees. Organizations can invest in various types of support to facilitate the adjustment process of PwCD by focusing on rebuilding a meaningful self. Support should focus on facilitating intentional identity work through, for example, coaching and counselling, as well as by offering group-based reflexive spaces as identity work opportunities. The identity work can be guided by the self-reflective reconstruction process proposed in this study, which may support PwCD to negotiate the tensions between the broken and the ideal self and to enable the reconstruction of a meaningful self.
